# Endostemonine I as a Multi-Target Inhibitor of Kaposi’s Sarcoma-Associated Herpesvirus Oncogenic Pathways: An Integrative Computational Study

**DOI:** 10.3390/medsci14020237

**Published:** 2026-05-04

**Authors:** Imran Sama-ae, Mollaya Daloh, Aman Tedasen, Siriruk Changrob, Monthon Lertcanawanichakul, Pattamaporn Kwankaew, Phenphitcha Issaro, Natthanicha Maidam, Nichakan Rattanapong, Nurul Auma, Mirfart Kaseng, Malatee Tayeh

**Affiliations:** 1 Department of Medical Technology, School of Allied Health Sciences, Walailak University, Tha Sala District, Nakhon Si Thammarat 80160, Thailand; imran.sa@wu.ac.th (I.S.-a.); aman.te@wu.ac.th (A.T.); lmonthon@wu.ac.th (M.L.); pattamaporn.kw@wu.ac.th (P.K.); phenphitcha.is@mail.wu.ac.th (P.I.); natthanicha.mi@mail.wu.ac.th (N.M.); nichakan.ra@mail.wu.ac.th (N.R.); nurul.au@mail.wu.ac.th (N.A.); mirfart.ka@mail.wu.ac.th (M.K.); 2 Center of Excellence Research for Melioidosis and Microorganisms (CERMM), Walailak University, Tha Sala District, Nakhon Si Thammarat 80160, Thailand; 3 Kapho Hospital, Kapho District, Pattani 94230, Thailand; monlaya.nee@gmail.com; 4 Research Excellence Center for Innovation and Health Products (RECIHP), Walailak University, Tha Sala District, Nakhon Si Thammarat 80160, Thailand; 5 Drukier Institute for Children’s Health, Weill Cornell Medicine, New York, NY 10021, USA; sic4001@med.cornell.edu; 6 Hematology and Transfusion Science Research Center (HTSRC), Walailak University, Tha Sala District, Nakhon Si Thammarat 80160, Thailand

**Keywords:** Kaposi’s sarcoma, *Streptomyce*, network pharmacology, molecular docking, molecular dynamics simulations

## Abstract

**Background/Objectives:** Kaposi’s sarcoma (KS) is an angioproliferative malignancy caused by Kaposi’s sarcoma-associated herpesvirus (KSHV), characterized by aberrant angiogenesis, chronic inflammation, and endothelial cell transformation. Given the multi-factorial nature of KS pathogenesis, strategies that simultaneously modulate multiple mo-lecular targets are considered more promising than single-target approaches. However, effective multi-target therapeutic agents for KS remain limited, prompting this study to employ an integrative in silico pipeline. **Methods:** An integrative in silico pipeline combining compound screening, target predic-tion, network pharmacology, Gene Ontology (GO) and Kyoto Encyclopedia of Genes and Ge-nomes (KEGG) enrichment, protein–protein interaction (PPI) analysis, molecular docking, and molecular dynamics (MD) simulations was employed. Streptomyces-derived metabolites were prioritized based on chemical diversity, annotation, and clinical prece-dent. Predicted targets were intersected with KS-associated genes, with hubs ranked by network topology. Docking and MD simulations evaluated binding affinity and stability. **Results:** Endostemonine I emerged as the top candidate, engaging nine of ten hub proteins, including EGFR, mTOR, PTGS2, SRC, PARP1, PPARγ, MAPK1, MAPK14, and ICAM1. Key nodes such as mTOR, PTGS2, PPARγ, and MAPK14 are central to KS-related an-gi-ogenesis, inflammation, and viral oncogenesis. GO and KEGG analyses revealed en-richment in kinase activity, cell adhesion, and PI3K–Akt/mTOR and MAPK signaling pathways. Docking indicated strong binding to mTOR, PTGS2, PARP1, PPARγ, and MAPK14, while MD simulations confirmed stable interactions for mTOR, PTGS2, PPARγ, and MAPK14. **Conclusions:** Collectively, these proteins represent high-confidence, druggable KS targets, with Endostemonine I as a promising multi-target scaffold. These findings highlight the therapeutic potential of Endostemonine I and warrant further validation through future in vitro and in vivo studies.

## 1. Introduction

Kaposi’s sarcoma (KS) is a complex angioproliferative neoplasm primarily driven by Kaposi’s sarcoma-associated herpesvirus (KSHV), also known as human herpesvirus 8 (HHV-8) [1]. Although KSHV infection is necessary for KS development, it is insufficient on its own; cofactors such as immunosuppression, chronic inflammation, and genetic susceptibility often contribute to tumorigenesis [2]. Histologically, KS is characterized by spindle-shaped endothelial cell proliferation, neovascularization, and inflammatory cell infiltration [3]. KS is classified into four subtypes—classic, endemic, iatrogenic, and epidemic (AIDS-associated)—each with distinct epidemiological and clinical profiles [4]. Endemic KS remains prevalent in Sub-Saharan Africa, often linked to early childhood KSHV transmission via saliva and influenced by socioeconomic and environmental factors [4]. Epidemic KS, associated with HIV infection, is an AIDS-defining illness that frequently presents with aggressive visceral involvement and poor prognosis [5]. Despite advances in HIV management through antiretroviral therapy (ART), KS treatment remains challenging due to delayed diagnoses, inconsistent immune reconstitution, and limited chemotherapy access in low-resource settings [6]. Notably, KS can develop even in individuals with well-controlled HIV and high CD4 counts, suggesting that persistent KSHV replication and heightened inflammation contribute to disease progression. Comorbid conditions such as KSHV inflammatory cytokine syndrome (KICS) may further exacerbate disease severity and complicate treatment [7].

The host immune system plays a central role in controlling KSHV infection through T-cell surveillance, interferon signaling, and natural killer (NK) cell activity. KSHV, however, evades these defenses via viral interferon regulatory factors (vIRFs), vFLIP, and viral microRNAs, which inhibit apoptosis, disrupt interferon pathways, and promote latency [1,3,8]. KSHV has a biphasic life cycle, alternating between latency and lytic replication. In KS lesions, latency predominates, with restricted gene expressions, including LANA, vFLIP, vCyclin, and Kaposin, that maintain episomes, inhibit apoptosis, and drive proliferation [2]. Reactivation to the lytic phase, initiated by the replication and transcription activator (RTA), induces full genome expression and virion production [1]. KSHV manipulates host signaling pathways, including PI3K–AKT–mTOR, JAK–STAT, EGFR–MAPK, and NF-κB, to promote survival, proliferation, and immune evasion [3,9]. For example, vGPCR activates MAPK and PI3K to drive angiogenesis and proliferation, while viral interleukin-6 (vIL-6) mimics host IL-6, activating STAT3 and fostering inflammation and tumor growth [8]. Additionally, the viral ubiquitin ligase K5 downregulates ICAM-1 and MHC-I, thereby reducing recognition by cytotoxic T cells and NK cells [8].

Given the inherent complexity of host–virus interactions, single-agent therapies directed at isolated molecular targets may prove insufficient. Network pharmacology, which prioritizes multi-target modulation within host–virus interactomes, provides a more integrative framework [10,11]. By leveraging biological databases such as KEGG and STRING, this approach identifies therapeutically relevant protein–protein interactions. Its effectiveness has already been demonstrated in diverse cancer contexts, such as tetrandrine influenced PI3K–Akt, mTOR, MAPK, and cell cycle pathways in breast cancer [12], curcuma-derived compounds acted on STAT3, EGFR, and HSP90AA1 in osteosarcoma [13], artesunate engaged PI3K/AKT/mTOR and p53 signaling in choroidal melanoma [14], and Neosetophomone B targeted fibroblast growth factor receptors across multiple cancer models [15]. Despite these promising precedents, applications in Kaposi’s sarcoma remain limited, highlighting the need for deeper investigation.

This study focuses on secondary metabolites derived from *Streptomyces* spp., prolific members of the *Actinobacteria phylum* renowned for producing chemically diverse and pharmacologically potent compounds [16]. Between 2015 and 2020, over 279 novel metabolites were isolated from *Streptomyces*, many exhibiting anticancer, antimicrobial, and enzyme-modulating activities [17]. These metabolites originate from both terrestrial and marine sources [17], and genome mining has revealed that individual strains often harbor numerous uncharacterized biosynthetic gene clusters [18]. Although their effects on KS-associated viral proteins have not yet been directly examined, structural and functional similarities to known modulators suggest potential interactions. This provides a strong rationale, grounded in pharmacological evidence and target-based drug discovery principles, for exploring these metabolites as antiviral and antitumor candidates in KS research. Accordingly, this study aims to: (i) evaluate drug-likeness, pharmacokinetic properties, and toxicity profiles of selected *Streptomyces*-derived metabolites using computational tools; (ii) identify putative KS-related targets through network pharmacology and protein–protein interaction analyses; and (iii) investigate molecular interactions between candidate ligands and these targets via molecular docking and molecular dynamics simulations. This integrative approach seeks to uncover novel compounds and propose mechanistic models supporting their therapeutic potential in KS treatment.

## 2. Materials and Methods

### 2.1. Streptomyces-Derived Natural Compounds Library Preparation

A comprehensive compound library was established from secondary metabolites produced by *Streptomyces* spp., a genus widely recognized for its prolific biosynthetic capacity. The selection process utilized the Natural Products Atlas 2.0, an open-access database that serves as a curated repository of microbial natural products [19]. Metabolites of *Streptomyces* origin were systematically selected from the Natural Products Atlas 2.0 and categorized by chemical class, biological activity, and structural data availability. The resulting library encompassed diverse compound groups, including polyketides, nonribosomal peptides, alkaloids, and hybrids, and provided the foundation for computational analyses such as drug-likeness evaluation, pharmacokinetic profiling, and target prediction to identify potential therapeutic candidates.

### 2.2. Drug-Likeness and Pharmacokinetics Evaluation

Drug-likeness and pharmacokinetic properties of the *Streptomyces*-derived compounds were evaluated following the protocol described in a previous publication [20] using SwissADME, a web-based tool for predicting pharmacokinetics, drug-likeness, and medicinal chemistry compliance of small molecules [21]. The assessment applied widely recognized drug-likeness filters, including Lipinski’s rule of five (Pfizer) [22], Ghose filter (Amgen) [23], Veber filter (GSK) [24], Egan filter (Pharmacia) [25], and Muegge filter (Bayer) [26], together with the Abbott bioavailability score [27] and Pan Assay Interference Compounds (PAINS) alerts [28]. Pharmacokinetic parameters analyzed encompassed gastrointestinal absorption, blood–brain barrier (BBB) permeability, P-glycoprotein substrate status, and cytochrome P450 (CYP) enzyme inhibition profiles. All natural compounds from the internal library were included in the evaluation. Exclusion criteria: Compounds were excluded if they exhibited excessive molecular size based on physicochemical parameters, including molecular weight > 500 g/mol, high heavy atom count, excessive rotatable bonds, or elevated topological polar surface area (TPSA). Additional exclusion criteria included poor gastrointestinal absorption, pass of blood–brain barrier permeability, P-glycoprotein substrate activity, or inhibitory activity toward CYP isoforms (CYP1A2, CYP2C19, CYP2C9, CYP2D6, CYP3A4). Compounds failing any drug-likeness filter, scoring a bioavailability index < 0.55, or triggering PAINS alerts were also excluded.

### 2.3. ADME and Toxicity Prediction

Toxicity profiles of *Streptomyces*-derived compounds were predicted using ADMETlab 3.0 [29], a machine learning–based platform for assessing absorption, distribution, metabolism, excretion, and toxicity (ADMET) properties. The evaluated toxicity endpoints included potential hERG channel blockades, drug-induced liver injury (DILI), human hepatotoxicity (H-HT), Ames mutagenicity, carcinogenicity, respiratory toxicity, and acute oral toxicity in rat models. Additional endpoints comprised neurotoxicity, ototoxicity, hematotoxicity, nephrotoxicity, genotoxicity, and violations of the FDA maximum recommended daily dose (FDAMDD). Only compounds that satisfied drug-likeness and pharmacokinetic criteria were subjected to toxic screening. Compounds predicted to exhibit any of the toxicological risks were excluded to ensure the selection of candidates with favorable safety profiles for subsequent computational analyses.

### 2.4. Target Prediction and Therapeutic Implications in Kaposi’s Sarcoma

Predicted protein targets of *Streptomyces*-derived compounds were identified using SwissTargetPrediction (2019 version) [30], which infers potential targets based on structural similarity to known bioactive ligands [31,32]. A cutoff of ≥0.05 is applied to exclude low-confidence, potentially spurious predictions while retaining targets with a meaningful likelihood of interaction. This threshold ensures biological relevance and improves dataset robustness by focusing on candidates suitable for downstream therapeutic analysis. To obtain Kaposi’s sarcoma (KS)-associated targets, the Human Gene Database [33] was queried using the term “Kaposi sarcoma.” Overlapping targets between the compound-predicted and KS-associated datasets were visualized with a Venn diagram generated via Interactive Venn (https://www.interactivenn.net/ accessed on 12 May 2025) [34]. The intersected targets were subsequently prioritized for their potential therapeutic relevance.

### 2.5. Gene Ontology (GO) and Kyoto Encyclopedia of Genes and Genomes (KEGG) Pathway Enrichment Analysis

To elucidate the functional significance of the identified targets, Gene Ontology (GO) and Kyoto Encyclopedia of Genes and Genomes (KEGG) pathway enrichment analyses were performed. GO provides a standardized framework for describing gene functions [35,36], whereas KEGG offers a comprehensive resource for understanding gene functions within biological pathways and networks [37,38]. Enrichment analyses were conducted using ShinyGO 0.82 [39], applying a false discovery rate (FDR) threshold of 0.05 to determine statistical significance [40]. GO results were visualized as bar plots, while KEGG pathway results were presented as bubble plots.

### 2.6. Protein–Protein Interaction (PPI) Network Construction

Protein–protein interaction (PPI) networks were generated using the STRING database [41], which integrates both experimentally validated physical interactions and predicted functional associations. The resulting interaction data were imported into Cytoscape version 3.10.4 [42], a widely used platform for network visualization and analysis. Within Cytoscape, topological parameters were examined to characterize network architecture and identify critical nodes. Key hub genes were determined using the cytoHubba plugin, applying the Maximal Clique Centrality (MCC) algorithm [43], a robust method for ranking nodes based on their centrality within densely connected sub-networks. This approach enabled the identification of highly connected genes that may play pivotal roles in disease-related pathways, thereby providing a foundation for subsequent functional enrichment and mechanistic analyses.

### 2.7. Molecular Docking Simulations

Molecular docking of *Streptomyces*-derived compounds with hub proteins was conducted using AutoDock 4.2 [44,45], a widely employed tool for predicting ligand–protein binding interactions. Three-dimensional ligand structures were retrieved from PubChem [46] and prepared following established protocols [47]. Prior to docking, the ligands were optimized in Open Babel [48] using the Universal Force Field (UFF). Energy minimization was performed with the conjugate gradient algorithm for 200 steps, applying a convergence threshold of 0.1 kcal/mol to ensure stable conformations. This preprocessing step enhanced structural accuracy and minimized steric clashes, thereby improving the reliability of subsequent docking simulations. Charge assignment was performed using the Gasteiger charge model and hydrogen atoms were added at physiological pH (7.4) to ensure accurate protonation states, followed by refinement of bond orders, torsional angles, and topology to improve structural reliability, after which the ligand was converted into AutoDock-compatible formats (PDB and PDBQT) using AutoDock 4.2. To validate the docking protocol, co-crystallized ligands from the three-dimensional structures of the target proteins were re-docked using the same computational procedure applied to the test compounds, and the resulting poses were compared with their experimentally determined crystallographic conformations to evaluate parameter accuracy. When co-crystallized ligands were unavailable, well-established inhibitors or reference ligands reported in the literature were employed as substitutes to ensure reliable benchmarking. This combined strategy confirmed that the docking workflow could reproduce native binding orientations and provided confidence in the robustness of the protocol for subsequent ligand evaluation.

Crystal structures of key disease-related proteins were retrieved from the RCSB Protein Data Bank (PDB) at resolutions below 3 Å to ensure structural accuracy [49]. Non-essential water molecules and co-crystallized ligands were removed using BIOVIA Discovery Studio 2024, after which hydrogen atoms were added, charges assigned, and atom types defined; the processed structures were saved in PDB format and subsequently converted to PDBQT using AutoDock [50]. Essential cofactors were retained during receptor preparation for targets in which they stabilize pharmacologically relevant binding sites.

Docking simulations were conducted in AutoDock4 (version 4.2.6) using the Lamarckian Genetic Algorithm with 50 GA runs and a population size of 200 to balance computational efficiency with thorough conformational sampling, while grid maps were generated with AutoGrid 4.2, as shown in Table 1. Binding affinities and conformations were analyzed, and molecular interactions were visualized in BIOVIA Discovery Studio [51]. Final docking outcomes were assessed based on binding energies, interacting residues, and bonding patterns, with optimal ligand conformations identified by the lowest binding energy values (kcal/mol) and affinities compared between Endostemonine I and reference ligands (Table 2). Protocol validation was performed by re-docking co-crystallized ligands from reference protein structures and comparing predicted poses with crystallographic conformations, while the docking score of Endostemonine I was benchmarked against the positive control of Kaposi’s sarcoma-associated targets.

### 2.8. Molecular Dynamics (MD) Simulations

Molecular dynamics (MD) simulations were performed using the Desmond module (Schrödinger) [52], following a modified protocol from previous reports [53]. Complexes of Endostemonine I with prioritized hub proteins, including PTGS2, MAPK14, PARP1, mTOR, and PPARγ complexes, were prepared at pH 7.0 using the Protein Preparation Wizard, which added hydrogens, assigned bond orders, rebuilt missing side chains and loops, optimized hydrogen-bond networks, and sampled water orientations. The OPLS4 force field [54] was applied, with systems solvated in orthorhombic TIP3P water boxes in a 10 Å × 10 Å × 10 Å and neutralized with 0.15 M Na^+^ and Cl^−^ ions. Energy minimization employed steepest descent [55] and LBFGS [56] algorithms, followed by equilibration in NVT and NPT ensembles. Production runs of 200 ns were conducted under NPT conditions at 310 K and 1.01 bar, with long-range electrostatics treated using the Smooth Particle Mesh Ewald (PME) method and Lennard–Jones interactions at a 9.0 Å cutoff. Trajectories were recorded every 200 ps (~1000 frames), and post-simulation analyses, including RMSD profiles, RMSF plots, ligand–protein contact maps, and timeline interaction analyses, were carried out using Desmond’s Simulation Interaction Diagram tool (Desmond, 2022) [52] and R (Version 4.5.1) [57], providing a comprehensive view of structural stability, conformational dynamics, and key interaction hotspots throughout the simulations.

### 2.9. Protein and Ligand Visualization

Two-dimensional interaction diagrams were generated using BIOVIA Discovery Studio Visualizer 2024 [50], while high-resolution three-dimensional visualizations of protein–ligand interactions were produced with Schrödinger software [52].

## 3. Results

### 3.1. Streptomyces-Derived Natural Compounds Library Preparation

A comprehensive catalog of secondary metabolites from *Streptomyces* species was curated using the Natural Products Atlas 2.0 database, resulting in the identification of 5758 distinct compounds across multiple species. This extensive chemical repertoire underscores the remarkable diversity of *Streptomyces* and highlights their significant potential as a valuable source for drug discovery (Supplementary Table S1).

### 3.2. Drug-Likeness and Pharmacokinetics Evaluation

A comprehensive evaluation of drug-likeness and pharmacokinetic properties was performed for all 5758 natural compounds in the in-house *Streptomyces*-derived library. Based on predefined criteria outlined in Section 2 (Materials and Methods), 245 compounds satisfied the selection thresholds. The screening process incorporated multiple established filters, including Lipinski, Ghose, Veber, Egan, and Muegge, to ensure compliance with drug-likeness rules and medicinal chemistry standards. In addition, predictive models indicated that the selected compounds exhibited favorable pharmacokinetic characteristics, such as high gastrointestinal absorption and the capacity to cross the blood–brain barrier. These properties highlight their potential bioavailability and therapeutic relevance, thereby justifying their inclusion in subsequent computational and experimental analyses (Supplementary Table S2).

### 3.3. Toxicity Prediction

An extensive in silico toxicity assessment was conducted using ADMETlab 3.0 to evaluate multiple safety-related endpoints for *Streptomyces*-derived compounds. The 238 compounds were excluded due to predicted toxicological liabilities, including hepatotoxicity, mutagenicity, and cardiotoxicity risks. By applying these rigorous selection parameters, only seven compounds satisfied all safety criteria and were subsequently advanced for target prediction and therapeutic relevance analysis (Supplementary Table S3). The compounds that successfully passed toxicity screening were nonactinic acid (NPA005190), (7E)-7-ethyl-4,10-dihydroxy-7-undecene-3,6-dione (NPA016169), 2-acetylamino-3-hydroxy-4-methyl-benzoic acid methyl ester (NPA023108), 2-methyl-8-hydroxybenzeneheptanoic acid (NPA032351), Myxofacycline E (NPA033232), Endostemonine E (NPA033591), and Endostemonine I (NPA033595). These compounds represent a refined subset of the original library, characterized by favorable safety profiles and potential suitability for further pharmacological investigation.

### 3.4. Target Prediction and Therapeutic Implications of Streptomyces-Derived Natural Compounds in Kaposi’s Sarcoma

**Figure 1:**
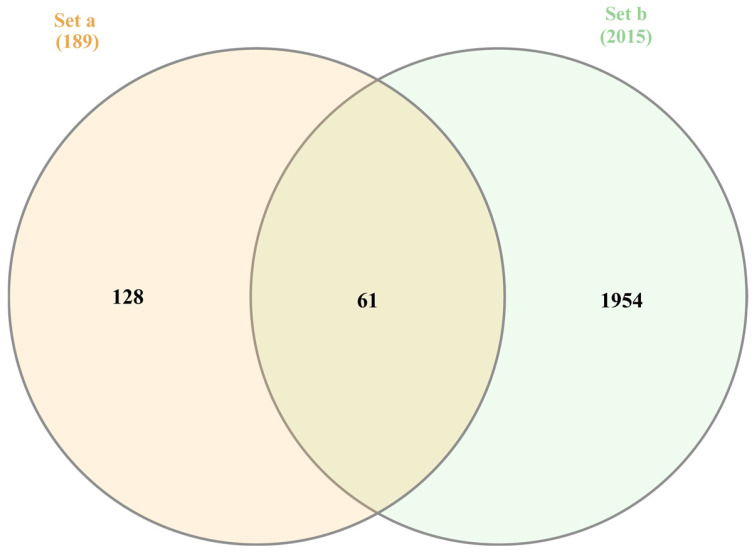
Venn diagram depicting the intersection between predicted targets of *Streptomyces*-derived natural compounds (Set a, *n* = 189) and Kaposi’s sarcoma (KS)-associated genes obtained from the GeneCards database (Set b, *n* = 2015). The overlap (*n* = 61) represents shared targets.

To identify potential therapeutic targets of *Streptomyces*-derived natural compounds, in silico target prediction was carried out using the SwissTargetPrediction web tool. Of the seven compounds that passed the toxicity evaluation described in Section 3.3, four yielded reliable target predictions and demonstrated acceptable probability scores. These included 2-acetylamino-3-hydroxy-4-methyl-benzoic acid methyl ester (NPA023108), 2-methyl-8-hydroxybenzeneheptanoic acid (NPA032351), Myxofacycline E (NPA033232), and Endostemonine I (NPA033595). The remaining three compounds, including nonactinic acid (NPA005190), (7E)-7-ethyl-4,10-dihydroxy-7-undecene-3,6-dione (NPA016169), and Endostemonine E (NPA033591), were excluded from further analysis due to either the absence of reliable predicted targets or insufficient structural similarity to active compounds in the reference database. Target prediction results revealed both overlapping and compound-specific protein associations. After removing duplicate entries across the four active compounds, a total of 189 unique protein targets were identified and retained for downstream analysis. In parallel, the GeneCards database was queried to compile 2015 genes associated with Kaposi’s sarcoma. Comparative integration of these datasets identified 61 intersecting genes, representing the overlap between compound-predicted targets and disease-related genes. This intersection highlights candidate molecular targets with potential therapeutic relevance and provides a focused subset for further mechanistic exploration. The overlap is illustrated in Figure 1 through a Venn diagram, which visually emphasizes the convergence between *Streptomyces*-derived natural compounds and Kaposi’s sarcoma-associated pathways. Collectively, these findings support the rationale for investigating the identified targets as mediators of antiviral and antitumor activity, thereby advancing the therapeutic application of *Streptomyces*-derived metabolites in KS research.

### 3.5. Gene Ontology (GO) Enrichment Analysis

**Figure 2:**
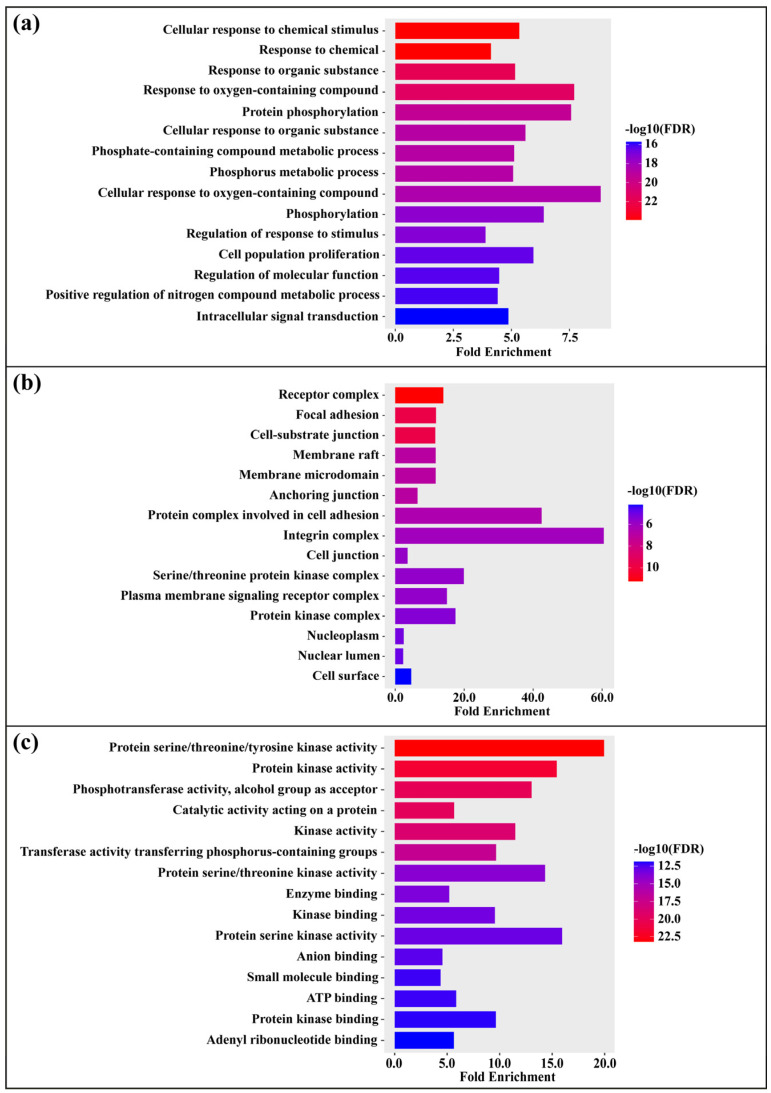
Gene Ontology (GO) enrichment analysis of the 61 intersecting target genes. The analysis categorized enriched GO terms into three domains: (**a**) Biological Process (BP), (**b**) Cellular Component (CC), and (**c**) Molecular Function (MF). The top 15 significantly enriched terms (false discovery rate [FDR] < 0.05) are shown for each category. Bar length represents fold enrichment, and color intensity indicates statistical significance (−log_10_[FDR]).

To elucidate the biological relevance of *Streptomyces*-derived compounds in the context of Kaposi’s sarcoma, Gene Ontology (GO) enrichment analysis was performed on the 61 candidate target genes using the ShinyGO version 0.82 platform. GO analysis organizes gene product annotations into three domains—Biological Process (BP), Cellular Component (CC), and Molecular Function (MF)—providing a structured framework for interpreting the functional roles of target proteins. This systematic approach enables the identification of biological activities and pathways potentially influenced by the selected compounds, particularly those implicated in viral oncogenesis, immune evasion, and angiogenesis associated with KS. The analysis revealed a large set of significantly enriched GO terms (false discovery rate [FDR] < 0.05), comprising 1351 entries: 1000 linked to biological processes, 112 to cellular components, and 239 to molecular functions (Supplementary File S4). The top 15 enriched terms for BP, CC, and MF are presented in Figure 2a–c, respectively, providing a visual overview of the functional landscape associated with the predicted targets (Figure 2). Collectively, these findings highlight the multifaceted biological roles of the candidate genes and underscore their potential mechanistic relevance in KS pathogenesis and therapy.

### 3.6. Kyoto Encyclopedia of Genes and Genomes (KEGG) Pathway Enrichment Analysis

**Figure 3:**
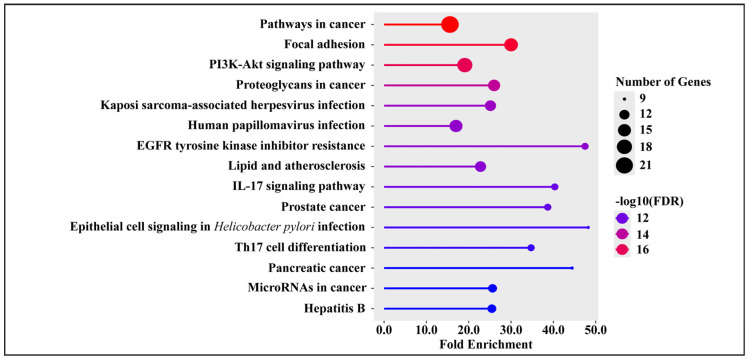
KEGG pathway enrichment analysis of 61 intersecting target genes. A total of 157 significantly enriched pathways were identified (false discovery rate [FDR] < 0.05). The figure illustrates the top 15 enriched KEGG pathways based on fold enrichment. Dot size reflects the number of associated genes, and color intensity represents statistical significance (−log_10_[FDR]).

To explore the signaling networks and molecular pathways potentially influenced by the candidate targets of *Streptomyces*-derived metabolites, KEGG pathway enrichment analysis was conducted using the ShinyGO version 0.82 platform. The analysis identified 157 significantly enriched pathways (false discovery rate [FDR] < 0.05), underscoring the broad involvement of these targets in diverse cellular and molecular processes (Supplementary Table S5). Among these, the top 15 pathways included cancer-related signaling, focal adhesion, the PI3K–Akt signaling pathway, proteoglycans in cancer, Kaposi’s sarcoma-associated herpesvirus infection, human papillomavirus infection, EGFR tyrosine kinase inhibitor resistance, lipid metabolism and atherosclerosis, the IL-17 signaling pathway, prostate cancer, epithelial cell signaling in *Helicobacter pylori* infection, Th17 cell differentiation, pancreatic cancer, microRNAs in cancer, and hepatitis B (Figure 3). Collectively, these enriched pathways highlight the multifaceted biological relevance of the predicted targets and provide mechanistic insights into how *Streptomyces*-derived natural compounds may exert therapeutic effects in Kaposi’s sarcoma and related malignancies.

### 3.7. Protein–Protein Interaction (PPI) Network Construction and Hub Gene Identification

**Figure 4:**
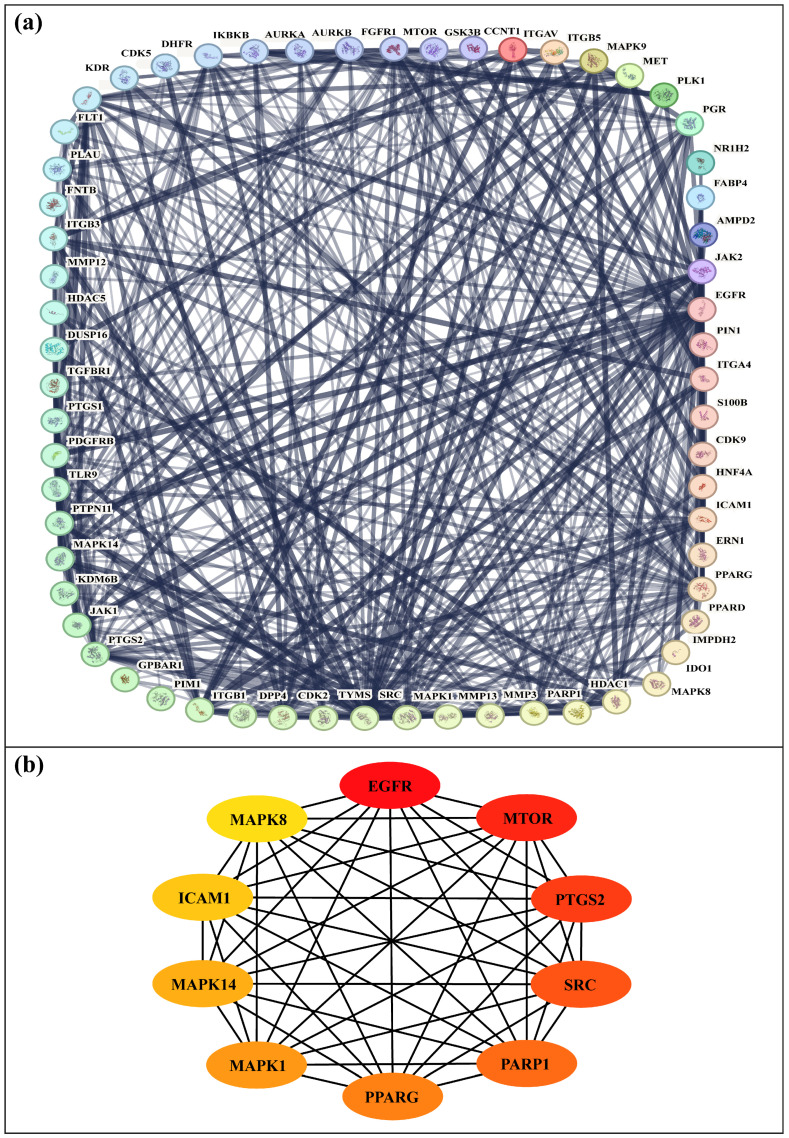
PPI network and hub gene analysis of 61 target genes. (**a**) Network constructed using STRING with a medium confidence score, where nodes represent proteins and edges indicate functional or physical associations. (**b**) Top 10 hub genes identified by cytoHubba using the MCC method in Cytoscape. Nodes are colored according to their MCC scores, where darker red indicates higher scores and lighter colors (yellow to orange) represent lower scores.

To elucidate the biological relevance of the predicted target proteins, a protein–protein interaction (PPI) network was constructed using the STRING database, incorporating the 61 candidate genes. The full STRING network option was selected to capture both functional and physical protein associations, with edges weighted according to interaction confidence scores. A medium confidence threshold was applied to ensure reliability while maintaining network inclusiveness. The resulting network was visualized in an interactive format (Figure 4a), enabling detailed exploration of connectivity patterns. Analysis revealed a densely interconnected set of targets, suggesting that many of these proteins participate in related biological processes and signaling cascades. Several proteins emerged as highly connected hub nodes, underscoring their potential central role in Kaposi’s sarcoma pathogenesis and highlighting them as promising candidates for further mechanistic investigation.

Key regulatory proteins within the network were identified using Cytoscape in conjunction with the cytoHubba plugin. To assess the relative importance of each protein, the MCC algorithm was applied, providing a robust ranking based on topological significance within the network. The ten highest-ranking hub genes were EGFR, MTOR (mTOR), PTGS2 (COX-2), SRC, PARP1, PPARG (PPARγ), MAPK1, MAPK14 (p38α), ICAM1, and MAPK8 (Figure 4b). These hub proteins, recognized as critical regulators of signaling pathways implicated in Kaposi’s sarcoma progression, were prioritized for molecular docking studies to evaluate their potential as therapeutic targets. This integrated approach, combining STRING-based network mapping with MCC-driven hub identification, enabled the systematic selection of high-impact molecular targets, thereby strengthening the rationale for subsequent in silico analyses and translational investigations.

### 3.8. Molecular Docking Simulations Between Streptomyces-Derived Compound and Hub Genes

To evaluate the binding affinities between bioactive compounds and key proteins implicated in Kaposi’s sarcoma, molecular docking simulations were performed. Rather than screening all candidate metabolites against the ten identified hub proteins, a targeted strategy was adopted, focusing on a ligand—Endostemonine I (NPA033595). This choice was informed by retrospective mapping of hub genes against compound–target prediction results generated by SwissTargetPrediction. Among the four *Streptomyces*-derived secondary metabolites assessed, Endostemonine I demonstrated the broadest target coverage, with nine of the ten hub genes, including EGFR, mTOR, PTGS2, SRC, PARP1, PPARγ, MAPK1, MAPK14, and ICAM1, predicted as potential binding partners. In contrast, 2-acetylamino-3-hydroxy-4-methyl-benzoic acid methyl ester (NPA023108) showed no overlap with the hub proteins, 2-methyl-8-hydroxybenzeneheptanoic acid (NPA032351) was associated with four proteins (PTGS2, PARP1, ICAM1, MAPK8), and Myxofacycline E (NPA033232) shared only one (PPARγ) (Supplementary Table S6). Given its extensive predicted relevance to KS-related molecular mechanisms, Endostemonine I was selected as the lead candidate for docking simulations, enabling a focused and systematic evaluation of its potential to modulate critical signaling proteins involved in disease progression.

**Figure 5:**
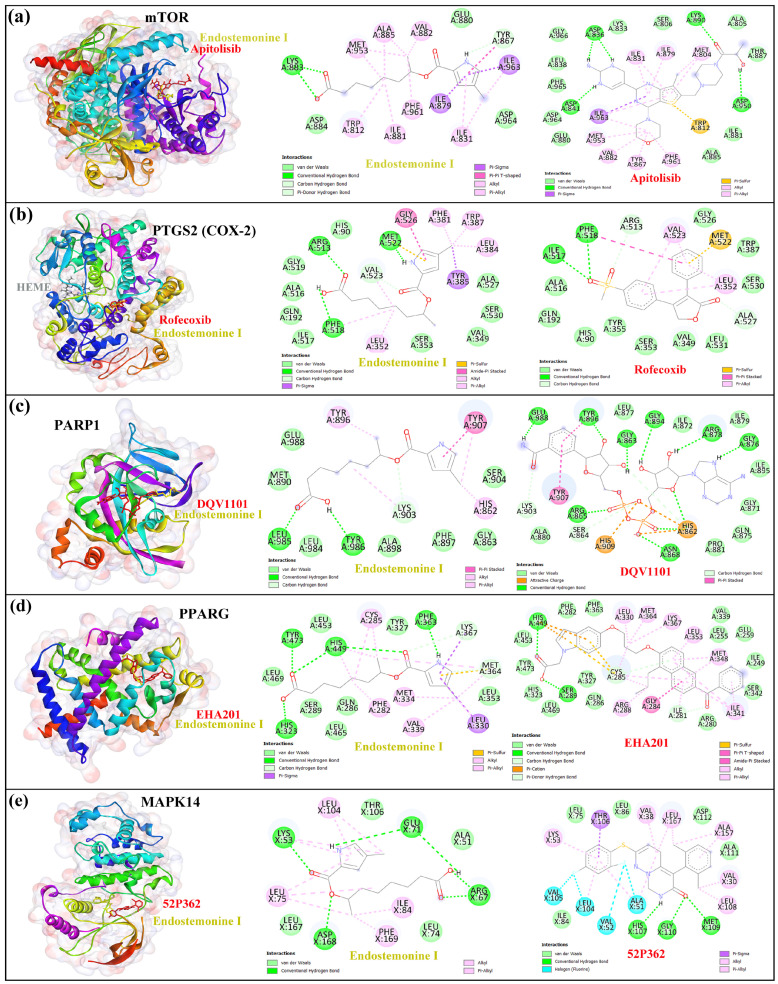
Two- and three-dimensional diagrams of Endostemonine-I with top target proteins. Left panels show overall protein structures with binding sites highlighted, and right panels display detailed ligand–protein interactions for (**a**) mTOR, (**b**) PTGS2, (**c**) PARP1, (**d**) PPARG, and (**e**) MAPK14.

Then, docking simulations were performed for Endostemonine I against nine hub proteins, including EGFR, mTOR, PTGS2, SRC, PARP1, PPARγ, MAPK1, MAPK14, and ICAM1. The results, summarized in Table 2 and Figure 5, report binding affinity values (kcal/mol) alongside detailed protein–ligand interaction profiles. Among these complexes, PTGS2, MAPK14, PARP1, mTOR, and PPARγ exhibited the lower binding energies than −7.0 kcal/mol, indicative of strong predicted interactions relative to the other targets. As shown in Table 2, the re-docking of co-crystallized ligands confirmed that the docking parameters reliably reproduced the native binding orientations of the positive controls. In addition, the docking score of Endostemonine I was compared with that of the co-crystallized ligand, further supporting the validity of the docking protocol.

In the three-dimensional structural view, Endostemonine I formed two hydrogen bonds with LYS883 and one with TYR867, along with several hydrophobic interactions at the same binding pocket occupied by apitolisib (GDC-0980), the co-crystallized ligand of the mTOR protein (Figure 5a). Within the Endostemonine I–PTGS2 complex, three hydrogen bonds were observed with ARG513, PHE518, and MET522, localized at the same active site as the co-crystallized reference ligand refecoxib (Figure 5b). For the Endostemonine I–PARP1 complex, binding occurred at the same pocket site as the DQV110 positive control, with hydrogen bonds formed to LEU985, TYR986, and LYS903 (Figure 5c). Similarly, Endostemonine I bound to PPARγ at the same site as EHA201, establishing hydrogen bonds with TYR473, HIS449, HIS323, and PHE363 (Figure 5d). As shown in Figure 5e, Endostemonine I also interacted with key amino acids of MAPK14, forming hydrogen bonds with LYS53, GLU71, ASP168, and ARG67 at the same pocket site as the co-crystallized ligand 52P362. However, docking provides only a static approximation of binding affinity and does not account for conformational flexibility, solvent effects, or dynamic stability under physiological conditions. To address these limitations, molecular dynamics (MD) simulations were subsequently conducted for the five most promising protein–ligand complexes. This analysis enabled evaluation of structural stability, conformational fluctuations, and interaction persistence within a simulated biological environment. By integrating docking with MD simulations, a more realistic and comprehensive assessment of Endostemonine I’s therapeutic potential was achieved, strengthening its candidacy as a modulator of pivotal proteins implicated in Kaposi’s sarcoma pathogenesis.

### 3.9. Molecular Dynamics Simulations

**Figure 6:**
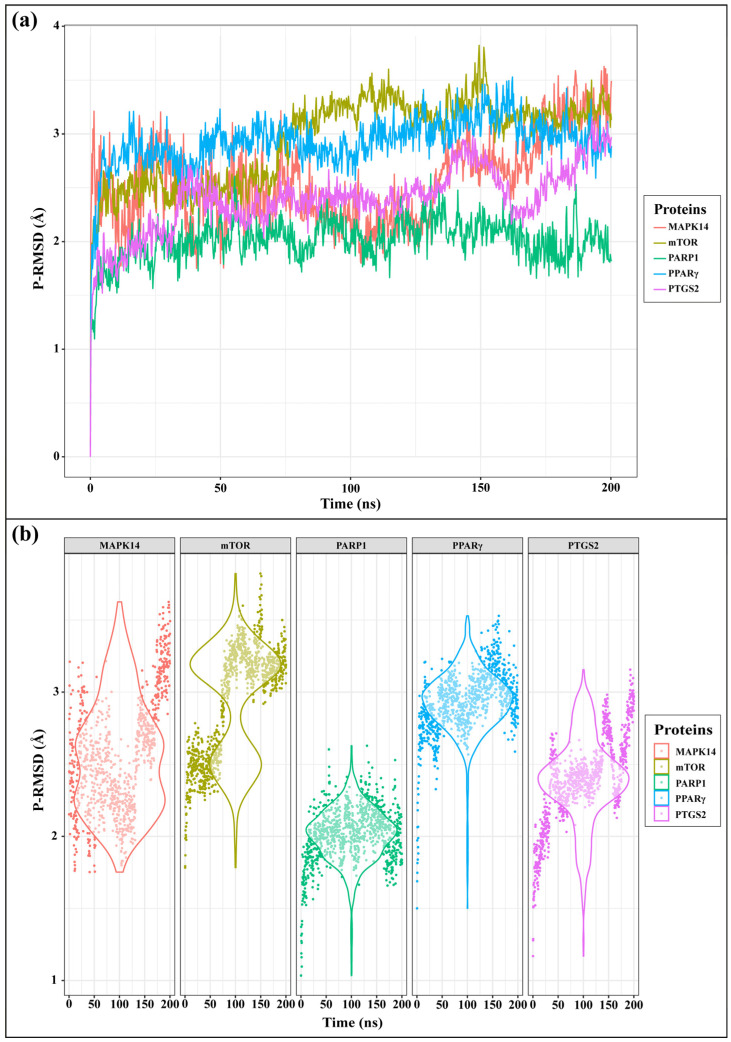
Protein root mean square deviation (P-RMSD) analysis of Endostemonine I complexes with mTOR, PTGS2, PARP1, PPARγ, and MAPK14 during 200 ns molecular dynamics simulations. (**a**) Line plots of P-RMSD trajectories illustrating backbone conformational changes over time. (**b**) Violin plots showing the distribution and variability of P-RMSD values.

To evaluate the stability of Endostemonine I bound to prioritized hub proteins (PTGS2, MAPK14, PARP1, mTOR, and PPARγ), 200 ns molecular dynamics (MD) simulations were performed. Trajectory analysis showed stable binding to PTGS2, MAPK14, and PPARγ, unstable retention in PARP1, and dual-site binding in mTOR (Supplementary Videos S1–S5). P-RMSD analysis confirmed equilibrium across all complexes, with mTOR and PPARγ showing moderate flexibility (~2.9 Å), MAPK14 consistent stability (2.55 Å), and PTGS2 and PARP1 lower deviations (2.39 Å and 2.01 Å), the latter being most stable (Figure 6a,b). L-RMSD analysis revealed target-dependent ligand stability (0.46–3.24 Å). MAPK14 was the most stable (mean 1.39 Å, narrow range), PTGS2 and PPARγ showed stable positioning (~1.6–1.8 Å), while mTOR and PARP1 exhibited greater fluctuations (up to 3.24 Å and 3.08 Å, respectively) (Supplement Figure S1).

**Figure 7:**
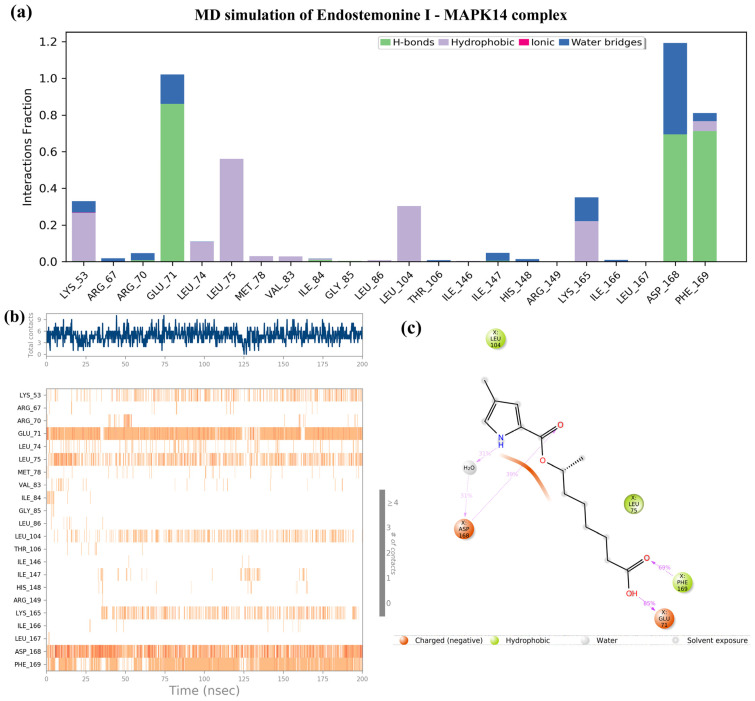
Molecular dynamics (MD) simulation of the Endostemonine I–MAPK14 complex over a 200 ns trajectory. (**a**) Timeline interaction analysis across the simulation. (**b**) Post-simulation contact maps depicting amino acid interactions. (**c**) 2D interaction diagram of Endostemonine I with MAPK14.

As shown in Figure 7, Endostemonine I emerged as the most promising candidate due to its strong binding affinity with key MAPK14 residues and high docking score, further validated by 200 ns MD simulations. Interaction fraction analysis revealed stable contacts with several key residues (Figure 7a,b). Hydrogen bonds and water-bridge interactions were dominant, particularly with GLU71, ASP168, and PHE169, while hydrophobic contacts were consistently observed with LYS55, LEU75, LEU104, and LYS165 (Figure 7a). Time-dependent analysis confirmed persistent interactions with GLU71, ASP168, and PHE169 throughout the trajectory, underscoring their importance in ligand recognition and binding stability (Figure 7b). The 2D interaction diagram further highlighted frequent hydrogen-bonding, hydrophobic, and polar contacts (Figure 7c). Collectively, these findings confirm the docking results and suggest that Endostemonine I engages MAPK14 through a combination of hydrogen bonds, electrostatic interactions, and water bridges, ensuring durable and favorable binding dynamics.

**Figure 8:**
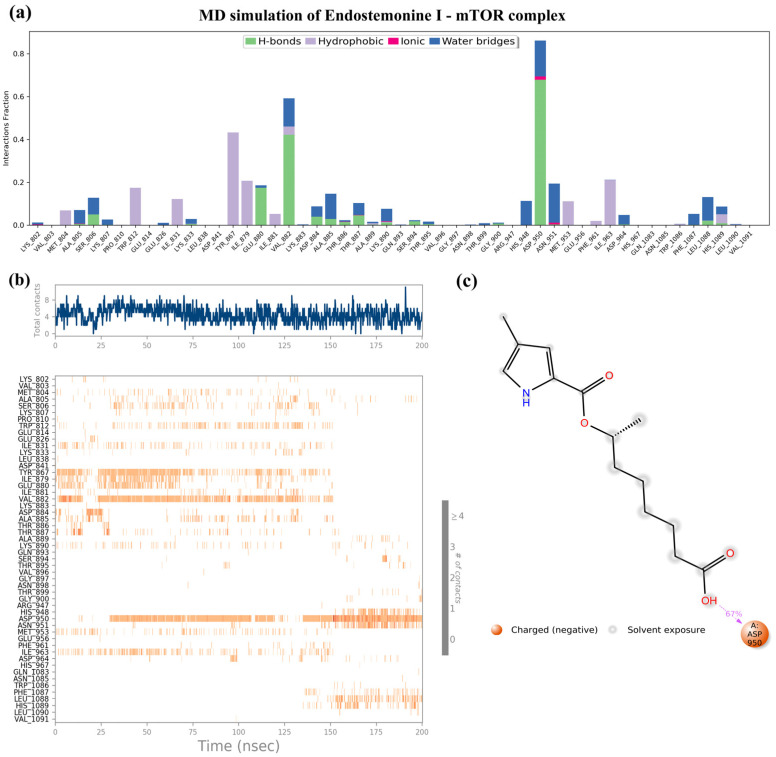
Molecular dynamics (MD) simulation of the Endostemonine I–mTOR complex over a 200 ns trajectory. (**a**) Timeline interaction analysis across the simulation. (**b**) Post-simulation contact maps depicting amino acid interactions. (**c**) 2D interaction diagram of Endostemonine I with mTOR.

As shown in Figure 8, Endostemonine I emerged as a promising candidate due to its strong binding affinity with key mTOR residues and high docking score, further validated by 200 ns molecular dynamics simulations. Interaction fraction analysis revealed transient contacts with several residues (Figure 8a,b). Hydrogen bonds and water-bridge interactions were observed, particularly with GLU880, VAL882, and ASP950, along with multiple hydrophobic contacts (Figure 8a). Time-dependent analysis showed that Endostemonine I maintained persistent interactions after 150 ns, with ASP950 exhibiting frequent and stable contacts between 150–200 ns, underscoring its role in ligand recognition and binding stability (Figure 8b). The 2D interaction diagram highlighted dominant electrostatic interactions with ASP950, showing the highest interaction frequencies (Figure 8c). Collectively, these findings confirm the docking results and suggest that Endostemonine I engages mTOR primarily through negative charge interactions, supporting durable and favorable binding dynamics.

**Figure 9:**
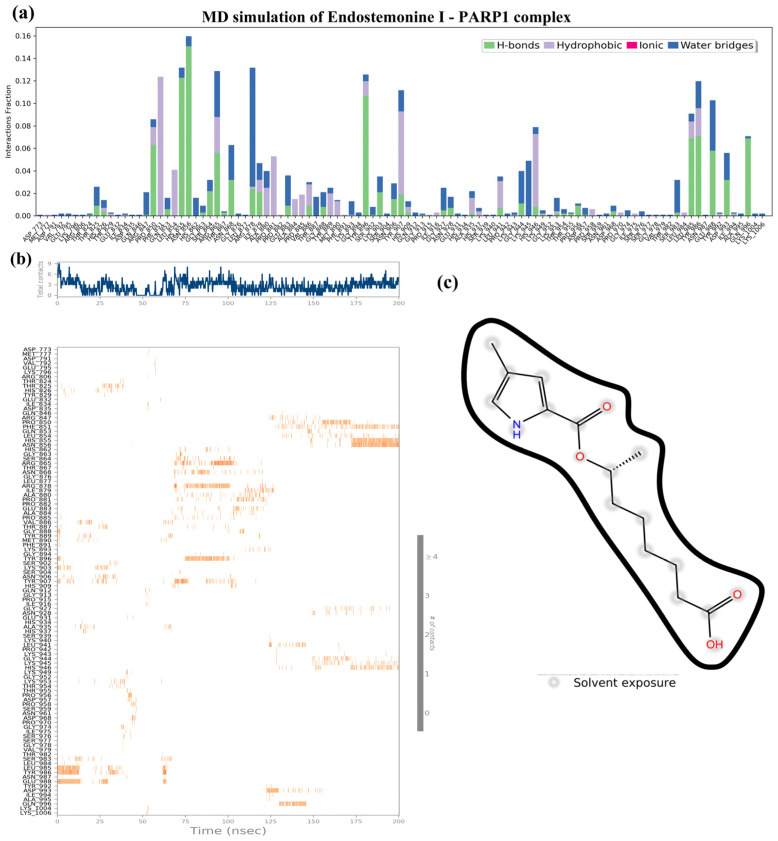
Molecular dynamics (MD) simulation of the Endostemonine I–PARP1 complex over a 200 ns trajectory. (**a**) Timeline interaction analysis across the simulation. (**b**) Post-simulation contact maps depicting amino acid interactions. (**c**) 2D interaction diagram of Endostemonine I with PARP1.

As shown in Figure 9, docking studies suggested strong binding of Endostemonine I to key PARP1 residues, but this was not supported by 200 ns molecular dynamics simulations. Interaction fraction analysis revealed only transient contacts after 175–200 ns (Figure 9a,b), with limited hydrogen bonds and water-bridge interactions observed. Time-dependent analysis indicated persistent interactions only late in the trajectory, primarily with HIS855 and ASN856 (Figure 9b). The 2D interaction diagram showed no consistent bonding, with solvent exposure dominating (Figure 9c). Collectively, these findings indicate that docking predictions were not aligned with MD results and suggest that Endostemonine I is unlikely to act as a PARP1 inhibitor.

**Figure 10:**
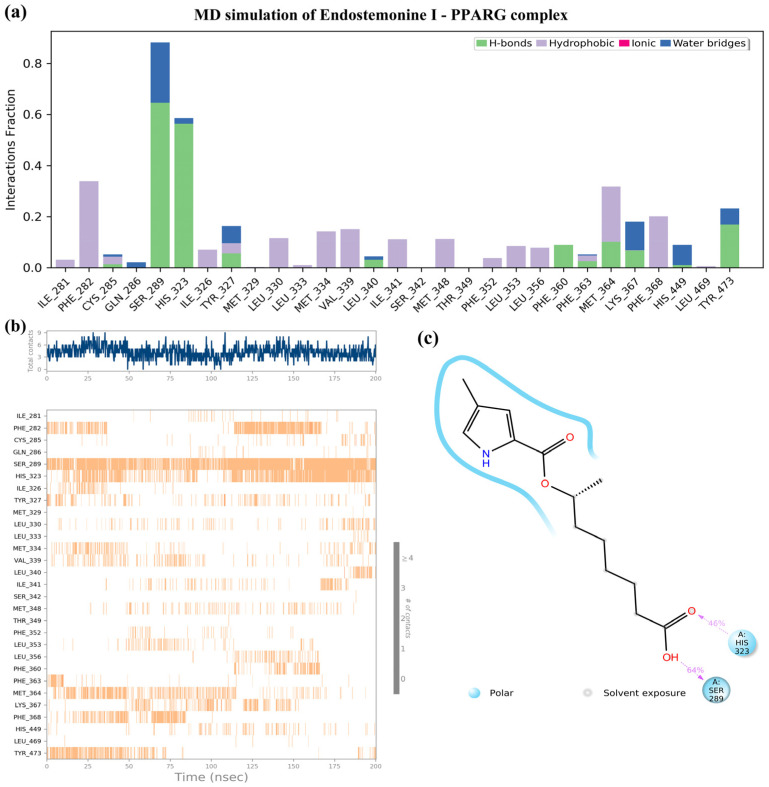
Molecular dynamics (MD) simulation of the Endostemonine I–PPARG complex over a 200 ns trajectory. (**a**) Timeline interaction analysis across the simulation. (**b**) Post-simulation contact maps depicting amino acid interactions. (**c**) 2D interaction diagram of Endostemonine I with PPARγ.

As shown in Figure 10, Endostemonine I emerged as a strong candidate due to its high docking score and strong binding affinity with key PPARγ residues, further supported by 200 ns MD simulations. Interaction fraction analysis revealed stable contacts with several residues (Figure 10a,b). Hydrogen bonds and water-bridge interactions were predominant, particularly with SER289, HIS323, and TYR473, while hydrophobic contacts were consistently observed with PHE282, MET346, and PHE368 (Figure 10a). Time-dependent analysis confirmed persistent interactions with SER289 and HIS323 throughout the trajectory, underscoring their importance in ligand recognition and stability (Figure 10b). The 2D interaction diagram further highlighted frequent polar interactions with SER289 and HIS323 (Figure 10c). Collectively, these findings validate the docking results and suggest that Endostemonine I engages PPARγ through hydrogen bonds and polar interactions, ensuring durable and favorable binding dynamics.

**Figure 11:**
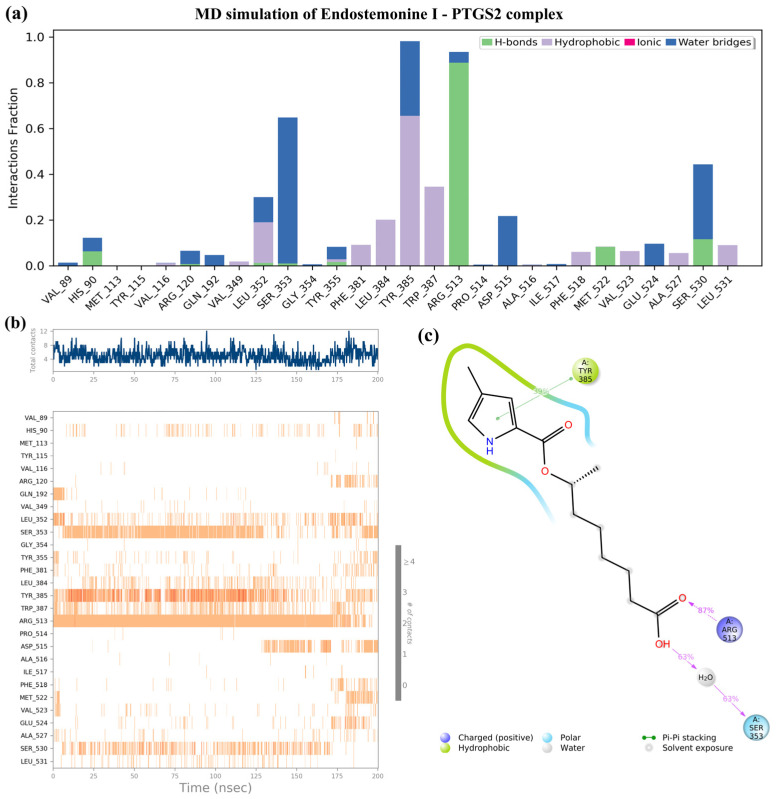
Molecular dynamics (MD) simulation of the Endostemonine I– PTGS2 complex over a 200 ns trajectory. (**a**) Timeline interaction analysis across the simulation. (**b**) Post-simulation contact maps depicting amino acid interactions. (**c**) 2D interaction diagram of Endostemonine I with PTGS2.

As shown in Figure 11, Endostemonine I emerged as the most promising candidate owing to its strong binding affinity with key PTGS2 residues and high docking score, further corroborated by 200 ns MD simulations. Interaction fraction analysis revealed stable contacts with several critical residues (Figure 11a,b). Notably, hydrogen bonding was observed with ARG513, while water-bridge interactions were predominant with SER353, TYR385, ASP515, and SER530. Hydrophobic contacts were consistently maintained with LEU352, LEU384, TYR385, and TRP387 (Figure 11a). Time-dependent analysis confirmed persistent interactions with SER353, TYR385, and ARG513 throughout the trajectory up to 175 ns, underscoring their importance in ligand recognition and binding stability (Figure 11b). Toward the end of the simulation, slight fluctuations were detected, disrupting some key interactions. The 2D interaction diagram further highlighted frequent positive charge, hydrophobic, and polar contacts (Figure 11c). Collectively, these findings reinforce the docking results and suggest that Endostemonine I engages PTGS2 through a synergistic combination of hydrogen bonds, electrostatic interactions, hydrophobic contacts, and water bridges, ensuring durable and favorable binding dynamics.

## 4. Discussion

In this comprehensive in silico study, combining compound screening, network pharmacology, molecular docking, and molecular dynamics, Endostemonine I emerged as a promising *Streptomyces*-derived metabolite with potential therapeutic relevance against KS. This conclusion is strengthened by consistent evidence across multiple analytical layers, including rigorous drug-likeness and toxicity filtering, disease-relevant target prediction, GO and KEGG pathway enrichment, PPI hub identification, and structure-based simulations.

We prioritized *Streptomyces*-derived metabolites for their exceptional chemical diversity, detailed annotation in open resources, and extensive clinical precedent. The Natural Products Atlas (NPAtlas) [19,58,59], an open-access, curated database of bacterial and fungal natural products, guided compound selection and contextualized chemotypes within established structural series. Its recent updates emphasize microbial sources and provide cross-linked metadata to support downstream computational analyses. Genomic studies reveal that *Streptomyces* strains harbor large inventories of biosynthetic gene clusters (BGCs), many of which remain silent or uncharacterized, ensuring a continuing supply of novel scaffolds. Comparative genome-mining across hundreds to thousands of genomes consistently reports high BGC counts per strain and broad class diversity (e.g., NRPS, PKS, terpenes, lanthipeptides) [60,61], underscoring the genus’s suitability for multi-target drug discovery. Historically and contemporarily, *Streptomyces* has yielded multiple frontline anticancer and immunomodulatory agents, such as doxorubicin from *S. peucetius* and the mTOR-pathway modulator sirolimus (rapamycin) from *S. hygroscopicus* [62,63,64], exemplifying both cytotoxic and signal-modulating pharmacologies relevant to modern multi-target strategies. Recent reviews and experimental surveys continue to highlight the anticancer and antiviral potential of *Streptomyces*-derived metabolites [16,65], reinforcing their value as chemotypes for network-informed screening. Collectively, comprehensive database coverage, genomics-driven novelty, and strong clinical precedent provide a robust foundation for selecting *Streptomyces* secondary metabolites for computational triage and mechanistic evaluation in KS.

We employed similarity-based target inference to nominate putative protein targets for *Streptomyces* metabolites that passed drug-likeness and toxicity filters. SwissTargetPrediction, which integrates 2D/3D similarity to bioactive ligands and ranks targets by probability, was selected for its retrained models and expanded bioactivity coverage in the 2019 update [30]. This approach may overlook novel or unconventional targets and can also yield false-positive associations when structural similarity fails to translate into functional relevance. Predicted targets were intersected with a curated list of KS-associated genes from the GeneCards suite, a compendium linking genes to diseases, pathways, and reagents [33], yielding 61 overlapping genes consistent with KS pathobiology. Components of the MAPK axis emerged prominently, aligning with evidence that KSHV activates ERK/JNK/p38 during primary infection and that p38 contributes to viral entry and lytic programs [66,67]. PTGS2 matched reports that KSHV, through vGPCR and vFLIP, induces COX-2/PGE2 signaling to sustain latency, inflammation, and angiogenesis [68,69]. Representation of the mTOR pathway further corresponded with evidence that rapalogs suppress KS growth by reducing VEGF output and constraining lytic replication [70,71].

To contextualize these genes, we constructed a STRING network and analyzed its topology in Cytoscape [41,42]. Hub nodes were ranked using cytoHubba’s Maximal Clique Centrality (MCC) algorithm, which reliably identifies essential network components [43]. Key hubs included EGFR, MTOR (mTOR), PTGS2 (COX-2), SRC, PARP1, PPARG (PPARγ), MAPK1, MAPK14 (p38α), ICAM1, and MAPK8, representing signaling nodes relevant to KS: pro-inflammatory and angiogenic drivers (PTGS2), stress-kinase signaling (MAPK14), nutrient-sensing growth control (mTOR), and receptor-proximal remodeling (EGFR/ICAM1). Literature supports these roles, with KSHV exploiting MAPKs for infection, vGPCR driving VEGF via the p38/MAPK–HIF-1α axis, COX-2/PGE_2_ sustaining latency, and mTOR activity targeted by rapamycin/everolimus [66,72,73]. In selecting Endostemonine I, we considered not only its predicted target overlap but also complementary criteria, including favorable binding affinity rankings, consistent ADMET properties, and structural uniqueness compared to other screened compounds, which collectively strengthened its candidacy as a multi-target scaffold.

Endostemonine I, predicted to engage 9 of 10 hub proteins, was selected for docking and molecular dynamics (MD) simulations based on a core principle of network pharmacology: prioritizing ligands that span high-centrality, disease-enriched nodes to test whether system-level relevance aligns with atomistic binding. Docking and MD analyses confirmed strong engagement of PTGS2, MAPK14, and PPARγ, as well as multi-site binding to mTOR. These findings are consistent with GO/KEGG enrichment of MAPK and PI3K–AKT/mTOR pathways and align with KS literature implicating PTGS2 [74], MAPK14 [66,72], PPARγ [75,76], and mTOR dependence [70,77].

We acknowledge several limitations. Similarity-based prediction may overlook novel targets lacking chemotype precedents and may bias toward well-studied classes. STRING integrates heterogeneous evidence, including indirect associations, while MCC ranks nodes by centrality rather than druggability. These considerations underscore the importance of combining enrichment and hub analyses with orthogonal structure-based evaluation and experimental validation, particularly for borderline nodes. By integrating SwissTargetPrediction, GeneCards mapping, and STRING/Cytoscape analytics, we derived a KS-relevant target ensemble that both reflects established KSHV biology and nominates tractable, multi-node hypotheses for wet-lab testing with Endostemonine I. This systems-to-structure continuity strengthens the rationale for prioritizing MAPK, PTGS2, PPARγ, and mTOR for validation in KS models.

GO enrichment analysis of the 61 genes using ShinyGO revealed significant clustering across Biological Process (BP), Cellular Component (CC), and Molecular Function (MF). The top 15 enriched terms (FDR < 0.05) are summarized in Figure 2a–c. Within BP, the most enriched terms—cellular response to chemical stimulus (~5.34×, *n* = 47), cellular response to organic substance (~5.61×, *n* = 39), protein phosphorylation (~7.57×, *n* = 34), and cell population proliferation (~5.95×, *n* = 34)—reflect enhanced signaling, metabolic responsiveness, and proliferative activity, all of which are elevated in KS driven by KSHV infection. KS tumors are highly vascularized, characterized by excessive angiogenesis, inflammatory infiltrates, and proliferation of infected endothelial (spindle) cells [78,79]. KSHV induces early and sustained VEGF-A and VEGF-C expression [80], and KS lesions consistently display marked spindle cell proliferation accompanied by angiogenesis and inflammation [81]. In CC, enrichment was observed for adhesion and signal transduction structures, including the integrin complex (~60.50×, *n* = 5), focal adhesion (~11.85×, *n* = 15), protein complex involved in cell adhesion (~42.46×, *n* = 6), membrane raft (~11.75×, *n* = 11), and receptor complex (~13.99×, n = 16). These structures are highly relevant to KS, where KSHV binds integrin receptors (e.g., α3β1) and activates focal adhesion kinase (FAK) to initiate viral entry and endothelial transformation [82]. In MF, kinase-related functions were enriched, including protein serine/threonine/tyrosine kinase activity (~19.95×, *n* = 25), protein kinase activity (~15.43×, *n* = 26), ATP binding (~5.87×, *n* = 26), and kinase binding (~9.56×, *n* = 21). This highlights phosphorylation-mediated signaling in KS progression, consistent with KSHV exploitation of MAPK and PI3K–Akt–mTOR pathways to promote proliferation and survival [67,83]. Phosphorylation-dependent regulation of viral and host proteins is also central to KSHV life cycle control via MAPK signaling [66,84].

KEGG analysis identified 157 enriched pathways (FDR < 0.05), with the top 15 including pathways in cancer (~15.57×, *n* = 22), focal adhesion (~30.01×, *n* = 16), PI3K–Akt signaling (~19.07×, n = 18), proteoglycans in cancer (~26.00×, n = 14), KSHV infection (~25.13×, *n* = 13), EGFR tyrosine kinase inhibitor resistance (~47.48×, *n* = 10), IL-17 signaling (~40.33×, *n* = 10), and Th17 cell differentiation (~34.73×, *n* = 10). The inclusion of KSHV infection and cancer pathways confirms direct relevance to KS biology, while the prominence of PI3K–Akt and focal adhesion pathways reflects KSHV’s strategy of hijacking endothelial signaling to drive angiogenesis and survival [83,85]. Together, these GO and KEGG results indicate that predicted targets of *Streptomyces*-derived compounds, particularly Endostemonine I, are enriched in processes central to KS hallmarks, including angiogenesis, proliferation, adhesion, and viral oncogenic signaling. These findings support the prioritization of kinase and adhesion network proteins for validation through docking and molecular dynamics simulations.

Molecular docking analysis revealed that Endostemonine I exhibited high-affinity interactions across five prioritized hub proteins (Figure 6), with PTGS2 and MAPK14 displaying the most negative binding energies, indicative of particularly strong predicted ligand–protein engagement (Table 2). PTGS2, a pro-inflammatory enzyme upregulated in KS lesions, promotes angiogenesis through prostaglandin E2 (PGE2) production [68]; high-affinity binding of Endostemonine I to PTGS2 suggests potential to attenuate KS-associated inflammation and angiogenesis. MAPK14, the predominant p38 isoform, plays a critical role in KSHV-mediated endothelial activation, VEGF induction, and cytokine production. KSHV vGPCR enhances VEGF via HIF-1α in a p38-dependent manner [72], and infection activates p38 along with ERK and JNK [66]. Pharmacological inhibition of p38 reduces KSHV-induced Ang-2 and cytokine output [86], while kaposin B activation of the p38/MK2 axis stabilizes cytokine mRNAs [87,88]. Docking with mTOR indicated slightly lower, yet still favorable, binding affinity. As KSHV activates the PI3K/Akt/mTOR pathway to support survival and angiogenic/metabolic reprogramming [83,89]—including through vGPCR- and K1-mediated PI3K–Akt signaling [90,91]—the multi-site retention of Endostemonine I in mTOR, further supported by MD simulations, suggests potential resilience to single-site perturbations. PPARγ exhibited moderate but significant docking scores; as a nuclear receptor, it modulates inflammation and angiogenesis, with agonists exerting anti-proliferative and anti-angiogenic effects [92,93]. Thus, PPARγ binding may contribute to multi-modal inhibition of KS progression. In contrast, PARP1 docking was less favorable, and subsequent MD simulations indicated unstable ligand association, suggesting it is a lower-priority target despite its role in gammaherpesvirus replication and latency [94]. Collectively, docking results highlight PTGS2, MAPK14, mTOR, and PPARγ as the most promising targets for Endostemonine I, offering multi-target modulation of angiogenesis, inflammation, and survival pathways central to KS pathobiology. These predictions warrant biochemical and cell-based validation.

Molecular dynamics simulations further evaluated the stability of Endostemonine I interactions with PTGS2, MAPK14, mTOR, PPARγ, and PARP1 over a 200 ns trajectory under simulated physiological conditions. RMSD profiles of protein backbones and ligand heavy atoms indicated stable, persistent interactions for PTGS2, MAPK14, and PPARγ, potential multi-site binding for mTOR, and unstable association with PARP1. Endostemonine I demonstrated strong and persistent binding to MAPK14, PPARγ, and PTGS2, dominated by hydrogen bonds, water-bridge, and hydrophobic interactions, with stability confirmed by 200 ns molecular dynamics simulations. These findings underscore its potential as a promising KS-relevant candidate through multi-target engagement of key signaling proteins. In contrast, Endostemonine I exhibited only transient binding to mTOR and PARP1 residues, suggesting lower stability and reduced prioritization for these targets. These findings underscore the importance of combining docking and MD simulations to refine target prioritization and assess druggability in multi-target candidates such as Endostemonine I.

The integration of compound selection, target prediction, network pharmacology, functional enrichment, docking, and molecular dynamics (MD) simulations enabled systematic prioritization of molecular targets most relevant to KS. Among the identified hubs, PTGS2, MAPK14, mTOR, and PPARγ consistently ranked highest across multiple analytical parameters. Endostemonine I demonstrated the broadest predicted target coverage, engaging nine of the ten hubs—PTGS2, MAPK14, mTOR, PPARγ, PARP1, EGFR, SRC, MAPK1, and ICAM1. Functional enrichment linked these proteins to key KS-related processes, including angiogenesis, inflammatory signaling, and viral oncogenesis. Docking simulations further supported their relevance, with PTGS2, MAPK14, mTOR, and PPARγ among the top five in binding affinity, while MD simulations confirmed their stability, with mean protein RMSD values below 3 Å (Table 2, Figure 6) and sustained ligand retention throughout 200 ns (Supplementary Videos S1–S3 and S5). In contrast, PARP1, despite strong docking scores, exhibited unstable ligand engagement in MD simulations (Supplementary Video S4) and was deprioritized. However, Kaposi’s sarcoma-associated herpesvirus (KSHV) also exploits angiogenin (ANG) to suppress p53 function and promote endothelial and PEL cell survival, with LANA-1 and ANG shown to colocalize, coimmunoprecipitate, and interact with p53 and Mdm2 in infected cells. Silencing ANG or blocking its nuclear translocation induces p53 activation, apoptosis, and loss of cell survival, underscoring its role in maintaining viral latency. Together, these findings suggest that therapeutic strategies targeting both canonical signaling hubs (PTGS2, MAPK14, mTOR, PPARγ) and ANG-mediated suppression of p53 may provide complementary avenues for disrupting KSHV latency and progression of KS [95].

Cross-validation of bioinformatic prediction, network topology, molecular recognition, and dynamic stability identifies PTGS2, MAPK14, mTOR, and PPARγ as high-confidence therapeutic targets for in vitro and in vivo validation. These proteins occupy central positions in KS-associated signaling networks and represent druggable nodes in other angioproliferative and inflammation-driven malignancies, making them attractive for multi-target therapeutic strategies. A potential falsification of this model would occur if Endostemonine I fails to reduce PGE_2_ output (PTGS2), phospho-p38 (MAPK14), or p-S6K/p-4EBP1 (mTOR) in KSHV-infected endothelial cells under predicted ADME conditions. Definitive experimental testing will therefore be essential to confirm or refute the therapeutic potential indicated by these in silico findings, using approaches such as kinase inhibition assays, endothelial cell proliferation assays, and KSHV-infected cell models under physiologically relevant conditions. These complementary experimental strategies will provide critical validation of computational predictions and establish the translational relevance of Endostemonine I as a multi-target candidate.

## 5. Conclusions

This study presents a comprehensive in silico framework that integrates compound selection, target prediction, network pharmacology, functional enrichment, molecular docking, and molecular dynamics simulations to identify and prioritize potential therapeutic targets for Kaposi’s sarcoma (KS). Endostemonine I, a *Streptomyces*-derived secondary metabolite, emerged as the top candidate, demonstrating multi-target potential against PTGS2 (COX-2), MAPK14 (p38α), MTOR (mTOR), and PPARG (PPARγ)—key nodes in KS-associated angiogenesis, inflammatory signaling, and viral oncogenesis. The convergence of bioinformatic prediction, systems-level network analysis, and atomistic modeling underscores the robustness of these findings and highlights Endostemonine I as a promising lead scaffold for further therapeutic development.

While these results highlight compelling therapeutic hypotheses, they remain predictive and require experimental validation. Future work should include biochemical assays to confirm binding, cell-based models to evaluate functional effects under KSHV infection, and pharmacokinetic/pharmacodynamic profiling to assess translational potential. Given the multi-target profile of Endostemonine I, its ability to modulate interconnected pathways may also be relevant to other angioproliferative and inflammation-driven malignancies. Expanding this integrative computational framework to broader chemical libraries and pathogen-specific networks could accelerate the discovery of novel multi-target therapeutics with enhanced clinical efficacy.

## Figures and Tables

**Table 1:** Details of the protein targets in the PDB database and the Grid docking parameters in molecular docking simulations.

Active Site Grid Maps for Molecular Docking Simulations									
Protein Name:	EGFR	MTOR	PTGS2	SRC	PARP1	PPARG	MAPK1	MAPK14	ICAM1
PDB ID:	1M17	3TL5	5KIR	1Y57	9DMC	2F4B	3W55	6SFO	1IAM
Current total grid Pts per map:	531,441	531,441	531,441	531,441	531,441	531,441	531,441	531,441	531,441
Number of points in x-dimension:	80.00	80.00	80.00	80.00	80.00	80.00	80.00	80.00	80.00
Number of points in y-dimension:	80.00	80.00	80.00	80.00	80.00	80.00	80.00	80.00	80.00
Number of points in z-dimension:	80.00	80.00	80.00	80.00	80.00	80.00	80.00	80.00	80.00
Spacing (Angstrom):	0.375	0.375	0.375	0.375	0.375	0.375	0.375	0.375	0.375
Center Grid Box: x-center	22.80	20.44	22.41	14.41	−18.72	6.31	13.32	1.77	33.46
Center Grid Box: y-center	2.08	62.44	−0.14	34.34	23.82	−7.20	39.41	0.33	75.72
Center Grid Box: z-center	52.95	21.18	32.96	41.94	37.18	37.69	44.01	−20.95	6.27

**Table 2:** Molecular docking simulations between Endostemonine I and 9 hub target proteins compared to co-crystalized positive control.

Protein Name	Common Name	PDB ID	Compound/Co-Crystalized Ligand or Drugs	Binding Affinity (kcal/mol)	Inhibition Constant
Epidermal Growth Factor Receptor	EGFR	1M17	Endostemonine I	−6.49	17.40 µM
			Erlotinib (co-crystallized)	−7.58	2.79 µM
Mechanistic Target of Rapamycin	mTOR	3TL5	Endostemonine I	−7.11	6.15 µM
			Apitolisib or GDC-0980 (co-crystallized)	−11.02	8.37 nM
Prostaglandin-Endoperoxide Synthase 2	PTGS2	5KIR	Endostemonine I	−7.68	2.33 µM
			Rofecoxib (co-crystallized)	−10.13	37.56 nM
SRC Proto-Oncogene, Non-Receptor Tyrosine Kinase	SRC	1Y57	Endostemonine I	−5.53	88.71 µM
			MPZ (co-crystallized)	−9.16	191.42 nM
Poly(ADP-Ribose) Polymerase 1	PARP1	9DMC	Endostemonine I	−7.37	3.39 µM
			DQV1101 (co-crystallized)	−6.76	11 µM
Peroxisome Proliferator-Activated Receptor Gamma	PPARγ	2F4B	Endostemonine I	−7.00	7.41 µM
			EHA201 (co-crystallized)	−11.88	1.94 nM
Mitogen-Activated Protein Kinase 1	MAPK1	3W55	Endostemonine I	−6.68	12.64 µM
			FR148083 (co-crystallized)	−8.20	973.43 nM
Mitogen-Activated Protein Kinase 14	MAPK14	3FC1	Endostemonine I	−7.61	2.66 µM
			52P362 (co-crystallized)	−9.32	147.87 nM
Intercellular Adhesion Molecule 1	ICAM1	1IAM	Endostemonine I	−5.97	42.28 µM
			Cam-IN-1 (PubChemID 10361323)	−6.26	25.94 µM

## Data Availability

The original contributions presented in this study are included in the article/Supplementary Materials. Further inquiries can be directed to the corresponding author.

## Supplementary Materials

The following supporting information can be downloaded at: https://www.mdpi.com/article/10.3390/medsci14020237/s1, or https://drive.google.com/drive/folders/1_5VqUltYhtcGWSndUUxFLP3kWYzVwo78?usp=sharing (accessed on 18 April 2026). Supplementary Table S1. Library of *Streptomyces*-Derived Secondary Metabolites from the Natural Products Atlas 2.0. Supplementary Table S2. Drug-Likeness and Pharmacokinetic Screening of *Streptomyces*-Derived Compounds. Supplementary Table S3. In Silico Toxicity Prediction of *Streptomyces*-Derived Compounds. Supplementary Table S4. Gene Ontology (GO) Enrichment Analysis of Predicted Targets Associated with Kaposi’s Sarcoma. Supplementary Table S5. Kyoto Encyclopedia of Genes and Genomes (KEGG) Pathway Enrichment Analysis of Predicted Targets Associated with Kaposi’s Sarcoma. Supplementary Table S6. Comparison of Predicted Targets of *Streptomyces*-Derived Metabolites with the Top 10 Hub Proteins Identified by STRING–CytoHubba PPI Analysis. Supplementary Video S1. Molecular dynamics simulations video of Endostemonine I inside the PTGS2’s binding site under in vivo mimic condition. Supplementary Video S2. Molecular dynamics simulations video of Endostemonine I inside the MAPK14’s binding site under in vivo mimic condition. Supplementary Video S3. Molecular dynamics simulations video of Endostemonine I inside the PPARγ’s binding site under in vivo mimic condition. Supplementary Video S4. Molecular dynamics simulations video of Endostemonine I inside the PARP1’s binding site under in vivo mimic condition.

## Abbreviations

The following abbreviations are used in this manuscript:

KS	Kaposi’s sarcoma
KSHV	Kaposi’s sarcoma-associated herpesvirus
GO	Gene Ontology
KEGG	Kyoto Encyclopedia of Genes and Genomes
PPI	Protein–protein interaction
MD	Molecular dynamics
HHV-8	Human herpesvirus 8
KICS	KSHV inflammatory cytokine syndrome
vIRFs	viral interferon regulatory factors
CYP	Cytochrome P450
DILI	Drug-induced liver injury
H-HT	Human hepatotoxicity
MCC	Maximal Clique Centrality
