# Supplementary material for: Endostemonine I as a Multi-Target Inhibitor of Kaposi’s Sarcoma-Associated Herpesvirus Oncogenic Pathways: An Integrative Computational Study
Source: Med Sci (Basel). 2026 May 4;14(2):237. doi: 10.3390/medsci14020237 (PMC13214889; doi:10.3390/medsci14020237)
Supplement: Supplementary file 1 [file medsci-14-00237-s001.zip › Supplement Figure S1.pdf]

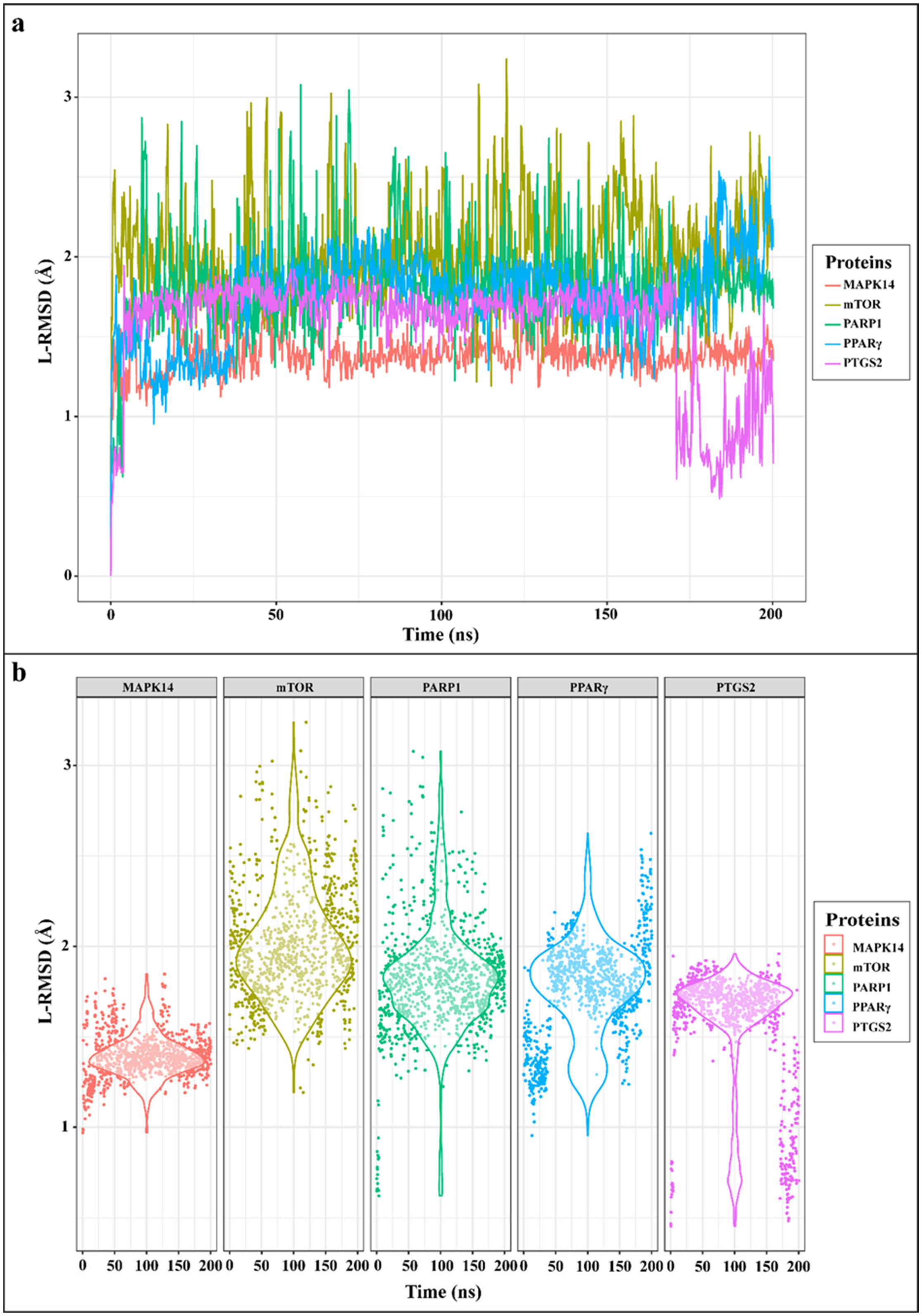

Supplement Figure S1. Ligand root mean square deviation (L-RMSD) analysis of Endostemonine-I complexes with hub proteins over a 200 ns molecular dynamics simulation using Desmond. (a) Line plots showing L-RMSD evolution for MAPK14, mTOR, PARP1, PPARγ, and PTGS2 complexes. (b) Violin plots summarizing L-RMSD distributions across the trajectory, indicating median and variation ranges for each complex.
